# Leveraging digital pathology and AI to transform clinical diagnosis in developing countries

**DOI:** 10.3389/fmed.2025.1657679

**Published:** 2025-09-25

**Authors:** Andrea C. del Valle

**Affiliations:** ^1^Department of Molecular Biosciences, The Wenner-Gren Institutet, Stockholm University, Stockholm, Sweden; ^2^Department of Physiology and Pharmacology, Karolinska Institutet, Solna, Sweden; ^3^Department of Microbiology, Tumor and Cell Biology, Karolinska Institutet, Solna, Sweden

**Keywords:** digital pathology, artificial intelligence, clinical diagnosis, machine learning, extensive data analysis, developing world

## Abstract

Computational pathology holds the promise of transforming the field of pathology by enabling faster and more accurate diagnosis and treatment planning. Digital pathology and artificial intelligence (AI) play a pivotal role in this transformation, offering more objective and precise diagnoses, increased efficiency, and the ability to handle large volumes of data. However, several translation barriers must be addressed to realize this potential, especially in developing countries. These barriers include the need for standardization of image acquisition and analysis, the limited availability of large, annotated image datasets, and a lack of computational expertise among pathologists. Overcoming these challenges requires collaboration among pathologists, computer scientists, and other experts, as well as the development of new technologies and algorithms. Despite these advancements, standardization and the creation of extensive annotated datasets remain critical issues. Addressing these barriers through collaborative efforts and technological innovation can significantly improve patient outcomes and reduce healthcare costs, making computational pathology a powerful tool in modern medicine in resource-limited settings.

## Introduction

Computational pathology has the potential to revolutionize the field of pathology by enabling faster, more accurate diagnosis and treatment planning. These datasets are designed to improve diagnostic efficiency, accuracy, and speed, while also aiming to reduce healthcare costs (1). Computational pathology methods are highly reproducible; this applies to well-known clinical cohorts such as the CAMELYON trial for lymph node metastasis in breast cancer (2) or the PANDA challenge for Gleason grading of prostate cancer (3). Clinical cohort studies, aided by computational pathology, have been implemented in Sweden to advance and predict prognosis in cancer patients, while also increasing scientific knowledge based on clinical evidence (4). Moreover, epidemiological studies leveraging computational pathology have enabled scientific discoveries that were previously unattainable. For example, recent research into placental changes associated with SARS-CoV-2 infection in pregnant women has benefited significantly from these advanced digital techniques (5). Sweden has also introduced the EXPRESSO (Epidemiology Strengthened by histoPathology Reports in Sweden) cohort, where epidemiologists can revise histopathology reports for a deeper clinical analysis (6). Projects led by Swedish institutions, such as the Center for Medical Image Science and Visualization (CMIV), have shown that digitizing pathology workflows can streamline case management, reduce manual handling, and lower overall administrative costs (7, 8).

Unfortunately, achieving this level of sophisticated technology and research output in resource-limited countries faces several translational barriers that must be overcome to realize this potential. In developing countries, professionals often have limited incentives to conduct research, and funding for research is scarce (9–11). In addition to brain drain, technological and methodological barriers hinder the implementation of digital pathology in developing countries. These barriers include the need for standardization of image acquisition and analysis, the limited availability of large, annotated image datasets, and the lack of computational expertise among pathologists (12, 13). Effectively addressing these challenges and increasing awareness among governments and institutions before implementation is essential for advancing research on a global scale. The successful adoption of computational pathology will depend on close collaboration between pathologists, computer scientists, and the ongoing development of innovative technologies and algorithms. Sustained communication among funding agencies, international research organizations, researchers, and pathologists (across both developed and developing countries) will be crucial to ensure coordinated progress and equitable access to these advancements (14). Through these efforts, computational pathology has the potential to improve patient outcomes and reduce healthcare costs, particularly in resource-limited settings (15). Unfortunately, since most research advancing digital pathology and AI is conducted in high-income countries, the earlier implementation of digital pathology in developing countries provides valuable feedback on the reality and direction of funding agencies in the future (16).

### Standardization in imaging acquisition

One significant barrier to the global application of digital pathology is the lack of standardization in image acquisition and analysis (17). Digital pathology images are generated using a variety of scanners and acquired at multiple resolutions, leading to significant variability in image quality. Furthermore, the application of diverse image analysis algorithms to these images can introduce additional inconsistencies in the extracted data. This lack of standardization poses substantial challenges for comparing results across studies and institutions, and hampers the development of robust, generalizable computational pathology algorithms (18). This challenge is even more pronounced in developing countries, where resources and access to advanced technology are often limited.

Nonetheless, in India, a collaborative effort between local hospitals and international research institutions has resulted in the development of standardized protocols for image acquisition and analysis (19–21). These institutions have produced high-quality, comparable data across different sites, utilizing uniform scanning equipment and standardized image analysis algorithms. This initiative has improved diagnostic accuracy and facilitated the development of robust computational pathology algorithms that can be applied in various settings (22). However, India’s booming tech scene positions it to tackle data privacy, infrastructure, and ethical concerns through AI research, data infrastructure upgrades, and strong ethical frameworks (23). India’s successful efforts in standardizing the acquisition of pathology images and facilitating research in this domain offer valuable guidance for pathologists seeking to advance established consensus practices by integrating AI and digital pathology. This progress also paves the way for companies and startups to develop more secure cloud storage solutions for pathological data, ensuring robust encryption and the protection of patient privacy on a global scale.

### The need for large, annotated datasets and infrastructure investment

A significant barrier to progress in computational pathology is the limited availability of large, annotated image datasets. Substantial volumes of high-quality data are essential for effective training and validating machine learning algorithms, yet such datasets remain scarce in developing countries. Certain endemic diseases would greatly benefit from extensive, population-specific annotated image collections. The annotation process itself is labor-intensive and often hampered by insufficient funding, especially in developing countries where resources for pathological research are limited. Data sharing is further constrained by privacy and ethical concerns. In addition, the lack of robust infrastructure and financial support in developing regions exacerbates the challenges associated with generating and disseminating large, annotated datasets.

Nevertheless, in Sub-Saharan Africa, a project funded by international health organizations has focused on creating large, annotated image datasets for training machine learning algorithms (24). The project has successfully created extensive datasets by actively engaging local pathologists in the annotation process and equipping them with targeted training and resources. These datasets now serve as a foundation for developing and validating AI algorithms specifically tailored to address regional disease profiles and healthcare challenges. In response to these needs and to strengthen the capacity for data science-driven health research and innovation across Africa, the National Institutes of Health (NIH) has launched the Common Fund initiative, Harnessing Data Science for Health Discovery and Innovation in Africa (DS-I Africa) (24). This project has produced comprehensive datasets that support the development and validation of AI algorithms specifically designed to address regional disease patterns and healthcare challenges. International initiatives and academic exchange programs, which bring together local and external researchers, represent promising strategies for alleviating the shortage of pathologists in developing countries. The use of secure servers that enable the uploading of pathology slides without compromising personal data can further facilitate the exchange and adoption of advanced technologies in these settings. Importantly, the challenges associated with data sharing, workforce shortages, and technology adoption are not confined to developing countries but are global issues impacting healthcare systems worldwide (25, 26).

### Empowering pathologists for the digital era

Finally, a third barrier to the widespread adoption of computational pathology is the limited computational expertise among pathologists. Although pathologists possess deep knowledge of disease biology, many lack the specialized skills or resources necessary to develop and implement computational pathology algorithms. This skills gap not only hinders pathologists from fully leveraging the capabilities of these advanced tools but also poses challenges for computer scientists seeking to design algorithms that align with clinical needs. In developing countries, the situation is further exacerbated by a shortage of trained professionals and restricted access to educational resources, which impedes the integration of computational pathology into clinical practice. Addressing this barrier will require targeted training programs and interdisciplinary initiatives aimed at enhancing computational literacy among pathologists, thereby fostering more effective collaboration and accelerating the adoption of innovative technologies in pathology (27). Another approach is to leverage scientific diasporas from developing countries to train and engage with national healthcare facilities. However, in such a case, the systematic bureaucracy will primarily depend on national initiatives (28, 29). Programs, including academic institutions and technology companies, might support and provide pathologists with the skills to develop and apply computational pathology algorithms. However, these initiatives must also solve the region’s healthcare needs by fostering collaboration between pathologists and computer scientists (26).

## Discussion

Although the barriers, such as data acquisition, data quality, and expert training, hinder the global implementation of computational pathology (30). Their impact is much greater in developing countries due to specific systemic challenges. Resource-limited settings often experience ongoing underfunding of healthcare and scientific research, which weakens the development of strong digital infrastructure necessary for reliable data collection and secure storage (31, 32). The widespread issue of “brain drain” exacerbates the shortage of skilled pathologists and technical staff, thereby slowing the adoption of advanced technologies and the growth of local expertise (28). Additionally, frequent internet and power outages, limited access to modern equipment, and a lack of large, annotated datasets create significant obstacles that are less severe or easier to overcome in high-income countries (33). Hence, as discussed in this manuscript, it is essential to collaborate with overseas institutions or implement training programs supported by open-source software platforms and shared protocols, which can broaden access to annotated datasets and thus close long-standing gaps in AI and digital pathology research.

Cultural issues and policies also play a role, such as limited government involvement and fragmented regulatory systems, which hinder collaboration among key stakeholders and delay progress in digital pathology (34). Additionally, the absence of established national standards for digital pathology and AI technologies can lead to inconsistent practices among institutions, reducing interoperability and making cross-site research more difficult (35). In many developing countries, the regulatory environment often cannot keep up with rapid technological progress, leading to unclear policies on data privacy, ethical use, and validation of AI tools (36). This uncertainty hampers both investment and international research collaborations and may limit access to global data repositories or shared platforms. Without strong academic-industry-government networks, knowledge sharing remains fragmented, preventing the creation of comprehensive research ecosystems.

Despite these challenges, emerging models of success are appearing, such as inter-hospital collaborations and international partnerships, exemplified by India’s standardized protocols for image acquisition and analysis, which showcase the potential for sharing expertise and resources. The Sub-Saharan Africa project has shown that the adoption of cloud-based solutions, implementation of national pilot programs, and capacity-building initiatives can also drive progress. Interdisciplinary initiatives that bring together clinicians, data scientists, and engineers are essential to foster mutual understanding and collaboration, ultimately accelerating technology adoption. Furthermore, leveraging scientific diasporas represents a promising strategy (28, 37). Experts from the diaspora can play a pivotal role in capacity building by providing mentorship, remote training, and knowledge transfer to local healthcare facilities. However, the success of such approaches heavily depends on coordinated national policies that facilitate diaspora engagement and streamline bureaucratic processes. Expanding these efforts while developing context-specific policy frameworks and investing in local workforce development will be crucial to fully realizing the benefits of AI-assisted digital pathology in developing countries.

Considering the points discussed above, Table 1 provides a comprehensive checklist to support the effective adoption of AI-assisted digital pathology in developing countries. The checklist emphasizes the importance of strategic collaboration among local institutions, international partners, and policymakers to foster innovation and secure vital support. Improving digital infrastructure, such as ensuring reliable internet access and implementing standardized imaging equipment, is crucial for maintaining consistent data quality and streamlining workflow efficiency. The framework also highlights the need for developing standardized protocols and enforcing strict quality control measures to ensure data interoperability and reproducibility. Additionally, building local capacity through targeted training programs and retention strategies is essential for fostering sustainable expertise and preventing brain drain. Ethical, legal, and regulatory considerations are addressed by advocating for policies tailored to the local context that align with international standards to encourage responsible AI deployment. Implementing pilot projects is recommended to test feasibility and guide the development of scalable, adaptable solutions suited for various clinical settings. Continuous stakeholder engagement and awareness campaigns are key to building trust and integrating digital pathology strategies into national healthcare systems. Lastly, securing long-term, sustainable funding is vital to support operational continuity and motivate ongoing investment, thereby enhancing diagnostic capabilities and improving healthcare outcomes in resource-limited settings. This structured approach offers a practical roadmap for overcoming the unique challenges faced by developing countries in adopting advanced computational pathology technologies.

**Table 1:** Recommended checklist for advancing AI-assisted digital pathology in developing countries.

	Recommended actions	Description
□	Forge Strategic Collaborations	Build interdisciplinary partnerships locally and internationally; engage funders and policymakers early; promote dialog among experts.
□	Strengthening Digital Infrastructure	Invest in reliable internet and power; acquire standardized scanners and imaging tools; implement secure, scalable data systems.
□	Implement Standardized Protocols	Develop consensus protocols for image acquisition and processing; ensure data interoperability; establish quality assurance frameworks.
□	Build Local Capacity and Expertise	Deliver targeted AI and pathology training; create career incentives to mitigate brain drain; enable knowledge exchange programs.
□	Address Ethical, Legal, Regulatory	Advocate for localized data privacy, consent, and AI validation policies, align with international standards, and educate stakeholders.
□	Pilot, Evaluate, and Scale	Conduct pilot studies to test workflows and technologies; refine processes based on feedback; develop scalable models for diverse settings.
□	Promote Stakeholder Engagement	Enhancing understanding of AI pathology benefits, fostering continuous stakeholder communication, and integrating initiatives into national health strategies.
□	Secure Long-Term Funding	Identify diverse funding sources; plan for ongoing costs; demonstrate clinical and economic value for sustained investment.

## Final remarks

This perspective highlights the transformative potential of computational pathology and AI in reshaping the field of pathology, particularly in developed countries. These technologies promise substantial improvements in patient outcomes and reductions in healthcare costs by enabling more rapid and accurate diagnoses as well as optimized treatment planning. Currently, computational models have enabled significant advances in clinical diagnostics and genomic interpretation (38). For instance, algorithmic analysis of underutilized complete blood count parameters has facilitated the prediction of anemia (39), offering insights beyond traditional laboratory assessments. Similarly, computational approaches have established correlations between hepatitis C virus sequence variations and the rate of progression to hepatic fibrosis in individual patients (40). In the realm of genomics, sophisticated tools such as PolyPhen and SIFT are employed to predict the pathogenicity of specific nucleotide variants, enhancing the accuracy of variant interpretation (41–44). The integration of these computational tools, alongside comprehensive data retrieval from resources such as dbSNP (45), ClinVar (46), ClinGen (47), COSMIC (48), and related databases, empowers pathologists and geneticists to assess the clinical significance more effectively (49). Moreover, these approaches facilitate the interpretation of novel variants discovered during clinical testing, enabling the extraction of meaningful insights at the population level. However, to fully realize these benefits, several critical barriers must be addressed, especially in resource-limited settings. Key challenges include the need for standardized protocols in image acquisition and analysis, the scarcity of large, annotated image datasets, and the limited computational expertise among pathologists. Achieving successful integration of computational pathology and AI into clinical practice will require robust interdisciplinary collaboration across geographic and professional boundaries. Recent adoption of AI-pathology in low-income countries has shown that there is a huge opportunity to add value to the non-profit sector, and partnerships with NGO experts can help ensure that theoretical advances in AI research translate into benefits for everyone. Through coordinated global efforts, it is possible to overcome these obstacles and unlock the full potential of these technologies, ultimately advancing patient care and health outcomes worldwide.

## Data Availability

The original contributions presented in the study are included in the article/supplementary material, further inquiries can be directed to the corresponding author.
